# Theranostics Using MCM-41-Based Mesoporous Silica Nanoparticles: Integrating Magnetic Resonance Imaging and Novel Chemotherapy for Breast Cancer Treatment

**DOI:** 10.3390/ijms25158097

**Published:** 2024-07-25

**Authors:** Indira C. B. Pires, Samia I. Shuchi, Braulio de V. A. Tostes, Dayane K. D. do N. Santos, William L. Burnett, Burke C. Leonce, Omar R. Harvey, Jeffery L. Coffer, Idio Alves de Sousa Filho, Petrônio Filgueiras de Athayde-Filho, Severino A. Junior, J. Michael Mathis

**Affiliations:** 1 Department of Chemistry, Federal University of Pernambuco, Recife 50670-901, PE, Brazil; indira.carolina@ufpe.br (I.C.B.P.); braulio.tostes@ufpe.br (B.d.V.A.T.); dayane.kelly@ufpe.br (D.K.D.d.N.S.); 2 School of Biomedical Sciences, Departments of Microbiology, Immunology, and Genetics and Pharmacology and Neuroscience, University of North Texas Health Science Center, Fort Worth, TX 76107, USA; samia.shuchi@unthsc.edu; 3 Department of Chemistry and Biochemistry, Texas Christian University, Fort Worth, TX 76109, USAb.leonce@tcu.edu (B.C.L.); omar.harvey@tcu.edu (O.R.H.); j.coffer@tcu.edu (J.L.C.); 4 Institute of Chemistry, Federal Rural University of Rio de Janeiro, Rio de Janeiro 23890-000, RJ, Brazil; idiofilho@ufrrj.br; 5 Department of Chemistry, Federal University of João Pessoa, João Pessoa 58051-900, PB, Brazil; athayde-filho@quimica.ufpb.br

**Keywords:** breast cancer, contrast agent, gadolinium, MCM-41, mesoporous silica nanoparticle, MIH 2.4Bl, MRI, theranostics

## Abstract

Advanced breast cancer remains a significant oncological challenge, requiring new approaches to improve clinical outcomes. This study investigated an innovative theranostic agent using the MCM-41-NH_2_-DTPA-Gd^3^⁺-MIH nanomaterial, which combined MRI imaging for detection and a novel chemotherapy agent (MIH 2.4Bl) for treatment. The nanomaterial was based on the mesoporous silica type, MCM-41, and was optimized for drug delivery via functionalization with amine groups and conjugation with DTPA and complexation with Gd^3+^. MRI sensitivity was enhanced by using gadolinium-based contrast agents, which are crucial in identifying early neoplastic lesions. MIH 2.4Bl, with its unique mesoionic structure, allows effective interactions with biomolecules that facilitate its intracellular antitumoral activity. Physicochemical characterization confirmed the nanomaterial synthesis and effective drug incorporation, with 15% of MIH 2.4Bl being adsorbed. Drug release assays indicated that approximately 50% was released within 8 h. MRI phantom studies demonstrated the superior imaging capability of the nanomaterial, with a relaxivity significantly higher than that of the commercial agent Magnevist. In vitro cellular cytotoxicity assays, the effectiveness of the nanomaterial in killing MDA-MB-231 breast cancer cells was demonstrated at an EC_50_ concentration of 12.6 mg/mL compared to an EC_50_ concentration of 68.9 mg/mL in normal human mammary epithelial cells (HMECs). In vivo, MRI evaluation in a 4T1 syngeneic mouse model confirmed its efficacy as a contrast agent. This study highlighted the theranostic capabilities of MCM-41-NH_2_-DTPA-Gd^3^⁺-MIH and its potential to enhance breast cancer management.

## 1. Introduction

Breast cancer is the most common cancer among women, affecting about 2.3 million annually and causing 700,000 deaths worldwide, representing 17% of all female cancer fatalities [1]. Early detection significantly enhances treatment outcomes [2]. Improvements in prognosis largely stem from advancements in diagnostic imaging and the development of new treatments [3].

Breast cancer encompasses various disorders with distinct histological and molecular profiles, classified into subtypes such as luminal A (hormone receptor-positive, low Ki-67), B (hormone receptor-positive, high Ki-67, negative HER2), triple-negative tumor, and tumor HER2-positive, each possessing unique genetic and clinical characteristics [4,5,6]. Luminal A cancers are hormone receptor-positive, show low Ki-67 protein levels and high estrogen and progesterone receptor expression, respond well to hormonal therapy, and are often prognostically favorable. Luminal B cancers are also estrogen receptor-positive but have higher Ki-67 levels and may overexpress HER2, generally indicating a poorer prognosis. Basal-like cancers are typically triple-negative, express basal/myoepithelial cell genes, and are aggressive with limited treatment options, leading to worse outcomes [7].

Several risk factors influence breast cancer susceptibility beyond genetic mutations. Hormonal factors such as early menstruation, late menopause, and nulliparity increase risk [8], as does exposure to hormone replacement therapy (HRT) [9]. Lifestyle factors like alcohol consumption, high-fat diets, and inactivity also elevate risk [10], along with environmental exposures like ionizing radiation and certain chemicals [11]. Understanding the multifaceted etiology of breast cancer requires a holistic approach that considers genetic, hormonal, lifestyle, and environmental influences.

Identifying genetic markers and risk factors highlights the necessity for early and precise diagnostic methods. In breast cancer, advancements in medical technology, such as mammography, ultrasonography, and Magnetic Resonance Imaging (MRI), are crucial for detecting the disease in its initial stages, significantly impacting prognosis and treatment management.

MRI is the most sensitive method for detecting breast cancer, with higher sensitivity than mammography and ultrasonography. A meta-analysis showed pooled sensitivities of 90% for abbreviated MRI (ABB-MRI) and 92% for full diagnostic protocol MRI (FDP-MRI) [12]. MRI is particularly effective for detecting tumors in dense breast tissue [13,14] and does not use ionizing radiation, making it safer for young women or those needing frequent exams. It can also detect multiple tumors within one or both breasts [15]. However, MRI has limitations, including high costs, longer examination times [16], potential for false positives, and contraindications with certain metal implants [17]. The decision to use MRI involves weighing these risks and benefits.

MRI for breast cancer detection frequently uses gadolinium-based contrast agents, which are administered intravenously to enhance lesion visibility. These agents accumulate in areas of increased vascular activity, common in solid tumors, including breast cancer, allowing for precise tumor evaluation and the detection of multifocal or multicentric lesions. In addition, they help monitor tumor response to treatments like neoadjuvant chemotherapy [18,19].

Chemotherapy for breast cancer utilizes cytotoxic drugs to target rapidly dividing cells, applicable before surgery as neoadjuvant therapy to shrink tumors or post-surgery as adjuvant therapy to clear residual cancer cells. Chemotherapeutic agents are categorized into alkylating agents, antimetabolites, anthracyclines, topoisomerase inhibitors, and microtubule agents, each disrupting cancer cell functions like DNA replication or cell division [20]. Treatment choice depends on the subtype, stage, biological markers associated with the tumor, and prior treatment responses. However, many agents suffer from low solubility and pharmacokinetic issues, and chemotherapy is challenged by multidrug resistance, severe side effects, and treatment resistance [21,22].

Mesoionic compounds, a unique class of heterocycles, offer broad medicinal potential with anti-inflammatory, analgesic, and anti-tumor properties due to their five-member heterocyclic ring and delocalized π electron system [23]. The mesoionic compound 2-(4-chlorophenyl)-3-methyl-4-(4-methylphenyl)-1,3-thiazolium-5-thiolate (MIH 2.4Bl) has shown significant in vitro growth inhibition in various breast cancer cell lines, including 4T1, MCF-7, and BT-20, but faces challenges with solubility that hinder in vivo administration [24]. Solving solubility and administration issues remains crucial for transitioning mesoionic compounds from preclinical studies to clinical use in breast cancer treatment [25,26].

The mesoionic drug MIH 2.4Bl is characterized by a 5-member heterocyclic ring with a sextet of π electrons associated with a positive charge and a negative charge residing on an accompanying anion [25,26]. This unique charge distribution enables effective interactions with biomolecules like DNA and proteins [23]. Despite its internal charge dynamics, it remains overall neutral, allowing cellular penetration and functioning as an intracellular anti-tumor agent. While MIH 2.4Bl has shown efficacy against various breast cancer cell lines with minimal impact on normal cells, the in vitro studies have used dimethyl sulfoxide (DMSO) as a solvent, which makes in vivo studies and applications unfeasible [24,27]. To advance clinical applications, using nanomaterials like mesoporous silica nanoparticles for drug delivery offers a promising approach [28].

Integrating nanotechnology into chemotherapy enhances drug delivery systems, highlighting the critical role of nanomaterials in improving solubility and efficient delivery of agents like MIH 2.4Bl. Nanomaterials such as mesoporous silica nanoparticles (MSNs) offer substantial surface area and volume for high therapeutic loading. Surface modifications of these nanomaterials enable conjugation with paramagnetic elements like gadolinium, enhancing their utility in MRI and other imaging techniques [29]. Thus, these nanomaterials facilitate dual theranostic roles in treating and diagnosing oncological diseases like breast cancer [30,31,32].

Since their first report in 1992, MSNs have become a prominent platform for breast cancer theranostics, integrating targeted therapy with advanced diagnostics. MSNs are valued for their high surface areas, ordered pore structures, excellent biocompatibility, and customizable particle and pore sizes. These features make MSNs ideal for delivering chemotherapeutics and conducting disease diagnostics. In addition, MSNs can be functionalized with drugs [33], medical imaging contrast agents [34], and biomolecules for targeted applications [22,35,36]. Recent studies have demonstrated the application of MSNs loaded with photosensitizing agents for photodynamic therapy [37] and radiosensitizers to enhance radiotherapy [38]. We have also demonstrated the potential of MSNs radiolabeled with 177Lu for SPECT imaging and radiotherapy using an HT-29 colon cancer preclinical model [39].

Silica nanoparticles are gaining traction in breast cancer theranostics, a field combining therapy and diagnostics to diagnose, treat, and monitor treatment simultaneously [40,41]. Their porous structure facilitates medication loading for controlled release at targeted sites, improving treatment efficacy and reducing side effects. In addition, these nanoparticles can carry contrast agents for imaging diagnostics like MRI [42] or computed tomography (CT) scans [43], allowing for effective tumor detection [44].

Mesoporous silica is designed with tailored pore structures for specific applications [45]. MCM-41 features a highly ordered hexagonal pore structure with uniform sizes ranging from 2 to 10 nanometers, providing a large surface area of 800 to 1200 m^2^ [46], and its hydroxyl-rich pore surfaces facilitate chemical modifications and interactions [47]. Compared to SBA-15, the smaller pores of MCM-41 offer greater drug- and diagnostic agent- loading capacity [48]. Recent studies have emphasized the potential of MCM-41 coated with iron oxide [49] or loaded with Gd^3+^ [50] for applications in cancer theranostics.

This study investigates the confluence of advanced MRI technology and nanotechnology in utilizing a theranostic platform for breast cancer centered on the novel MCM-41-NH_2_-DTPA-Gd^3+^-MIH nanocomposite. It aims to address the pivotal challenges in breast cancer treatment and diagnosis, offering a paradigm shift towards more effective, personalized therapeutic strategies. By integrating MRI-based imaging with a novel chemotherapy agent into a single delivery platform, this research aspires to demonstrate its potential preclinical application in breast cancer as an approach to managing this formidable disease.

## 2. Results

### 2.1. MCM-41 Synthesis

**Figure 1:**
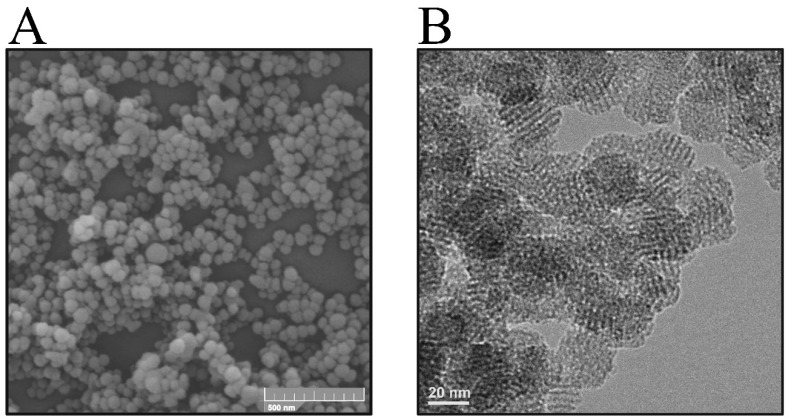
(**A**) Representative images of the synthesized MCM-41 nanomaterial were obtained by scanning electron microscopy (SEM) at a magnification of 160,000× and (**B**) transmission electron microscopy (TEM) at a magnification of 120,000×.

Initially, silica-surfactant micelles were formed, serving as critical templates for nanoparticle structuring. These templates guided the arrangement of silica molecules, facilitating the creation of a defined porous network. This process was followed by hydrolysis and subsequent condensation of the silica, culminating with the removal of the surfactant through calcination to obtain the final composition of the nanoporous material. Figure 1 shows the morphology of the synthesized nanomaterial.

A visual analysis of the micrograph shown in Figure 1A demonstrates the formation of nanoparticles with a uniform, spherical morphology. The particle size distribution of the mesoporous silica nanoparticles was calculated from the SEM images to be 60 ± 3.8 nm.

The micrograph in Figure 1B provides a visualization of one of the most distinctive features of the MCM-41-type silica nanomaterial: its highly ordered pore structure. The image showed a uniform pattern of dots and lines, representing a regular arrangement of the mesopores. This pattern highlighted a hexagonally packed order, demonstrating the long-range structural property of the nanomaterial. In addition, the image showed the presence of uniform, well-defined cylindrical pores throughout the synthesized nanomaterial, consistent with a precise order achieved during synthesis. These data confirm the structural characteristics of the nanomaterial, which are critical for its application involving the process of adsorption and controlled release of drugs [46,51,52].

**Figure 2:**
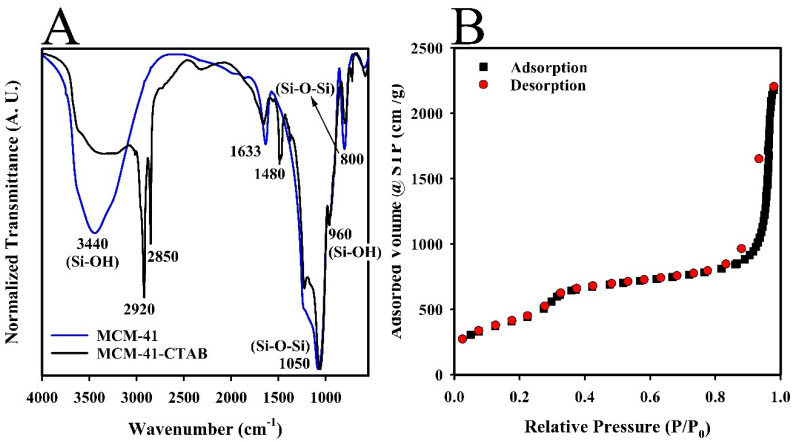
(**A**) FTIR analysis comparing unmodified MCM-41 (blue line) and CTAB-modified MCM-41-CTAB (black line) (**B**) N_2_ adsorption (■) and desorption (●) isotherms of the MCM-41 nanomaterial.

Fourier Transform Infrared Spectroscopy (FTIR) was employed to analyze the chemical structure of both MCM-41 and MCM-41-CTAB nanoparticles, as depicted in Figure 2A. The FTIR spectra revealed several distinctive absorbance bands indicative of the composition of the silica network. A broad and intense band at 3440 cm^−1^ was attributed to O-H stretching and axial vibration of silanol (Si-OH) groups on the silica surface, as well as bridged hydroxyl groups and water molecules physically adsorbed to the nanomaterial.

Additional bands were identified in the MCM-41 spectra. First, a band at 960 cm^−1^, corresponding to the asymmetric vibrations of silanol groups, was identified. Second, a band at 800 cm^−1^, corresponding to the symmetric stretching of siloxane (Si-O-Si) bonds, was noted, which is integral to the spherical structure of the silica. Third, a strong band at 1050 cm^−1^ was observed, associated with asymmetric axial vibrations of the Si-O-Si groups. Another significant band at 1632 cm^−1^ represented the bending vibrations of adsorbed water molecules [51,53,54].

Figure 2A also includes the FTIR spectra for the MCM-41-CTAB nanomaterial, which showed distinct bands at 2920 and 2850 cm^−1^, corresponding to the stretching modes of CH(CH_3_) and CH(CH_2_) functional groups from the CTAB surfactant, respectively. A notable band at 1480 cm^−1^, related to the bending modes of these groups, indicated the presence of CTAB. Post-thermal calcination, the disappearance of the bands at 1480, 2920, and 2850 cm^−1^ in the MCM-41-CTAB spectra (Figure 2A) confirmed the effective removal of the surfactant, demonstrating the changes in chemical composition due to the calcination process [55]. The results of this spectral analysis provide crucial insights into the structural and compositional dynamics of the synthesized nanoparticles.

Porosimetry analysis of the synthesized MCM-41 nanomaterial was conducted using nitrogen adsorption–desorption isotherms at 77 K, as depicted in Figure 2B. This analysis was essential for assessing the porosity characteristics of the nanomaterial. The resulting curves displayed the variations in the amount of nitrogen adsorbed by one gram of the silica surface across a relative pressure range from 0.0 to 1.0 at a constant temperature. The configuration of the isotherm was classified as type IV, which is characteristic of nanomaterials with mesoscopic porosity, as identified by the classification of the International Union of Pure and Applied Chemistry (IUPAC) [56]. The isotherm profile indicated a sequential three-phase adsorption process: initial single-layer and multilayer adsorption, followed by condensation within the capillaries of the nanomaterial, and culminating in multilayer adsorption on the external surface of the nanomaterial [55,57].

No hysteresis was observed in the initial desorption phase, a typical feature of MCM-41-type silica nanomaterials. However, a minor hysteresis loop observed in the subsequent desorption phase was attributed to the obstruction of pores at points where nanoparticles interconnected [52]. Using the DFT (density functional theory) method [58], the surface area of the nanomaterial was determined to be 1725 m^2^/g, along with a pore volume of 3.1 m^2^/g and an average pore size of 3.7 nm. These results demonstrated significant porosity and structural efficiency of the MCM-41 nanomaterial.

### 2.2. Functionalization of MCM-41 with APTES (MCM-41-NH_2_)

**Figure 3:**
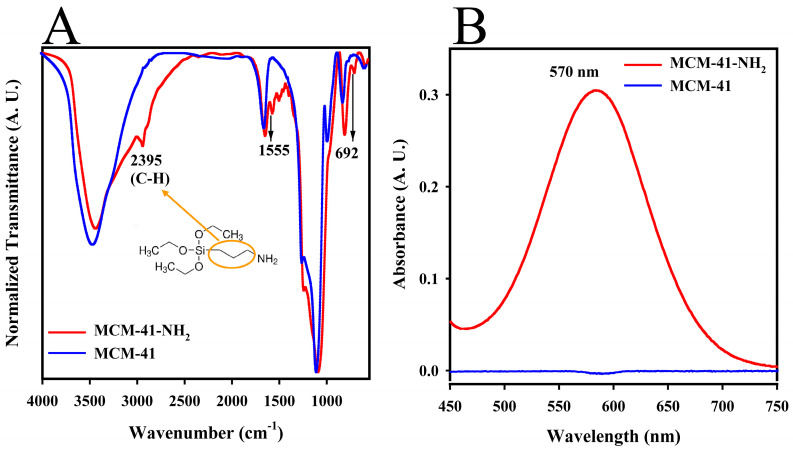
(**A**) FTIR spectra of unmodified MCM-41 (blue line) and amine-functionalized MCM-41-NH_2_ (red line). Illustration of C-H bonds is shown in the orange circle. (**B**) Absorbance spectra from a colorimetric assay using the ninhydrin reagent, demonstrating the presence of primary amino groups on MCM-41-NH_2_ (red line) compared to unmodified MCM-41 (blue line).

A comparison of the FTIR spectra between MCM-41 and MCM-41-NH_2_, shown in Figure 3A, revealed significant changes post-functionalization. Notably, a new absorption band at 2935 cm^−1^ appeared in the MCM-41-NH_2_ spectrum (red line), corresponding to C-H group vibrations from the propyl substituent of the APTES reagent [59]. In addition, bands at 1555 and 692 cm^−1^ were observed, indicative of the vibrations of the amine (N-H) groups introduced by APTES. These results demonstrated the successful functionalization of the silica surface for further conjugation with the APTES reagent [60,61].

A colorimetric assay using the ninhydrin reagent was conducted to verify the presence of amine groups on the MCM-41-NH_2_. The absorbance spectra shown in Figure 3B highlight the reaction outcomes, where only MCM-41-NH_2_ (red line) exhibited a distinct absorbance peak at 570 nm. This peak confirms the reaction of ninhydrin with the free amino groups on the MCM-41-NH_2_, leading to a visible color change from transparent to purple. The reaction mechanism involves a nucleophilic substitution where the amino group replaces the OH groups in the ninhydrin, followed by complex reactions that culminate in the formation of Ruhemann’s purple, a dark purple compound [62,63].

### 2.3. Conjugation of MCM-41-NH_2_ with DTPA (MCM-41-NH_2_-DTPA) and Complexation of MCM-41-NH_2_-DTPA with Gd^3+^ (SiO_2_-NH_2_-DTPA-Gd^3+^)

**Figure 4:**
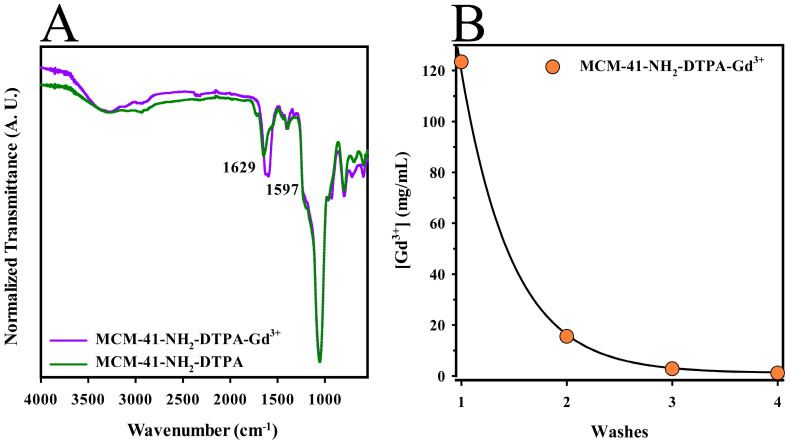
(**A**) FTIR spectra displaying changes post-conjugation of MCM-41-NH_2_ with DTPA (purple line) and its complexation with Gd^3+^ (green line) (**B**) Gadolinium ion concentrations in the MCM-41-NH_2_-DTPA-Gd^3+^ nanomaterial (●) measured by ICP-OES analysis after four sequential washes.

FTIR was employed to verify the chemical modifications and confirm the successful attachment of functional groups during the conjugation process. As shown in Figure 4A (green line), absorption bands at 1737 and 1648 cm^−1^ were noted, corresponding to the stretching vibrations of carbonyls (C=O). These bands likely originated from amide groups formed by the conjugation of DTPA with the terminal amine groups on the silica surface, or possibly from free carboxylic acids groups [64]. In addition, another characteristic DTPA band at 1395 cm^−1^, attributed to the symmetrically coupled stretching of the C-O bonds in carboxylate anions, was identified [65]. These observations indicated the successful conjugation of DTPA onto the silica surface.

Following the formation of the MCM-41-NH_2_-DTPA-Gd^3+^ complex, as shown in Figure 4A (purple line), shifts in the original carbonyl bands to lower frequencies were observed, now centered at 1629 and 1597 cm^−1^. These shifts indicated the presence of ionized carboxylate groups (-COO^−^) and were consistent with the coordination of these groups with Gd^3+^ ions. The reduction in frequency during this coordination process was likely due to the resonance structure of the carboxylates, which assumes characteristics closer to a single bond, influenced by the metal ions that reduce vibration frequency in this region [44,66].

The MCM-41-NH_2_-DTPA-Gd^3+^ nanomaterial underwent four washing cycles to remove excess Gd^3+^ ions, as unchelated gadolinium is toxic to human tissues [67]. Inductively Coupled Plasma Optical Emission Spectrometry (ICP-OES) analysis was used to determine the concentration of Gd^3+^ in each wash, as shown in Figure 4B. The concentrations of Gd^3+^ decreased significantly across the washes, from 123.4 mg/L in the first wash to 1.1 mg/L in the fourth, indicating efficient removal of excess ions. The final suspension of the nanomaterial, at a concentration of 1200 mg/L, exhibited a gadolinium ion concentration of 79.4 mg/L, confirming the presence of Gd^3+^ within the nanomaterial.

### 2.4. Changes in Zeta Potential of Nanomaterials following Surface Modifications

The zeta potential of the synthesized nanomaterials was measured to assess their surface charge and stability in suspension, as this affects their interaction with biological systems and their overall performance in applications such as drug delivery, imaging, and therapeutics. As shown in Table 1, the zeta potential values measured for the mesoporous silica nanoparticles showed significant variations after each modification. The MCM-41 exhibited a zeta potential of −22.5 mV, indicative of a predominantly negative surface, due to the presence of silanol groups on the surface of the mesoporous silica. After functionalization with amine groups, the zeta potential of the MCM-41-NH_2_ became positive, reaching a value of +12.9 mV. This shift to a positive value was due to the introduction of protonated amine groups (NH^3+^) on the surface, which neutralized the negative charge and imparted a net positive charge. The MCM-41-NH_2_-DTPA, which was conjugated with the DTPA ligand, showed a zeta potential that was negative again, equal to −16.1 mV. The conjugation with DTPA introduced additional negative carboxylate groups (COO^−^), which overwhelmed the positive amine groups and resulted in a net negative charge. These results confirm that each modification step effectively altered the surface chemistry of the nanoparticles.

### 2.5. Adsorption and Release of MIH 2.4Bl onto the MCM-41-NH_2_-DTPA-Gd^3+^ Nanomaterial

**Figure 5:**
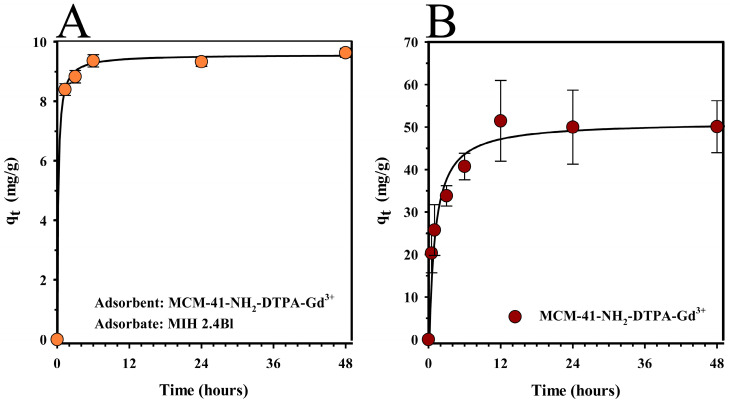
(**A**) Adsorption kinetics (●) and (**B**) release profile (●) of the mesoionic drug MIH 2.4Bl from the MCM-41-NH_2_-DTPA-Gd^3+^ nanomaterial as a function of time. Each data point represents the mean ± standard deviation of three replicates.

Adsorption assays were conducted to determine the equilibrium time of the system involving the MCM-41-NH_2_-DTPA-Gd^3+^ nanomaterial and the mesoionic drug MIH 2.4Bl. Figure 5A illustrates the drug adsorption kinetics, representing the amount of drug adsorbed per unit mass of the adsorbent (q_t_) over time. The assay utilized 10 mg of the nanomaterial as the adsorbent and 1.5 mL of a 3 mM methanol solution of MIH 2.4Bl as the adsorbate. The time points sampled in the study were 0.0, 1.3, 3.0, 6.0, 24.0, and 48.0 h.

From the graph analysis (Figure 5A), it was observed that there was rapid adsorption at the beginning of the process in the quantity of MIH 2.4Bl incorporated into the MCM-41-NH_2_-DTPA-Gd^3+^. However, over time, the adsorption capacity of the nanomaterial reached equilibrium. This effect was likely related to the saturation process of the nanomaterial, in which the adsorption gradually became slower due to the reduction of the quantity of available active sites. The maximum adsorption time was 6 h to produce MCM-41-NH_2_-DTPA-Gd^3+^-MIH, in which the adsorption capacity was equivalent to 9.3 mg of adsorbate per gram of adsorbent, representing a drug loading around 15% of the initial adsorbate concentration.

The MCM-41-NH_2_-DTPA-Gd^3+^-MIH nanomaterial, after adsorption, was also characterized by ICP-OES to determine the concentration of chelated gadolinium ions. In this experiment, a suspension of MCM-41-NH_2_-DTPA-Gd^3+^-MIH at a concentration of 1200 mg/L exhibited a gadolinium ion concentration equivalent to 73.3 mg/L.

A drug release study of MCM-41-NH_2_-DTPA-Gd^3+^-MIH was performed to determine the bioavailability of MIH 2.4Bl from the nanoporous nanomaterial (Figure 5B). In this experiment, a rapid release was observed in the first 12 h, reaching approximately 40% of the total drug amount. This initial pattern suggested that a significant fraction of the drug was less strongly retained, possibly in the more superficial regions of the nanomaterial or in pores with easier access. Subsequently, the release profile stabilized, showing a gradual release profile that reached a maximum of about 50% release in 48 h. This kinetic pattern suggested that the remainder of the drug was more deeply integrated into the porous structure or retained by stronger chemical interactions with the functional groups on the nanomaterial’s surface.

### 2.6. In Vitro Cytotoxicity Studies

**Figure 6:**
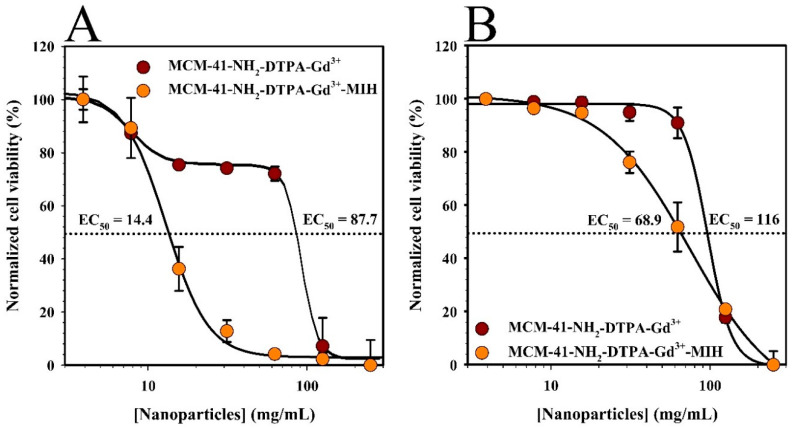
MTT assay results showing the cell viability of (**A**) MDA-MB-231 breast cancer cells and (**B**) normal human mammary epithelial cells (HMECs) after 96 h of treatment with increasing concentrations of MCM-41-NH_2_-DTPA-Gd^3+^ (●) and MCM-41-NH_2_-DTPA-Gd^3+^-MIH (●) nanoparticles. The dashed lines represent 50% normalized cell viability. Each data point represents the mean ± standard deviation of three replicates.

MTT assays were conducted to assess the cytotoxicity of the synthesized nanoparticles (MCM-41-NH_2_-DTPA-Gd^3+^ and MCM-41-NH_2_-DTPA-Gd^3+^-MIH) on the MDA-MB-231 breast cancer cell line and normal human mammary epithelial cells (HMECs). In these experiments, the cells were exposed to increasing concentrations of the nanoparticles (from 3.9 µg/mL to 250 µg/mL) for 96 h. The results shown in Figure 6A demonstrated a dose-dependent cytotoxic effect on the MDA-MB-231 cells, with a significant reduction in cell viability observed at lower concentrations of the MIH 2.4Bl drug-loaded nanoparticles compared to nanoparticles alone. In contrast, the HMECs (Figure 6B) exhibited higher viability across all concentrations, indicating selective cytotoxicity towards cancer cells. The half-maximal effective concentration (EC_50_) was determined to be significantly lower for the MDA-MB-231 cells (12.6 mg/mL) compared to HMECs (68.6 mg/mL), demonstrating selective cytotoxicity towards cancer cells.

### 2.7. Relaxometry and MRI Phantom Studies of MCM-41-NH_2_-DTPA-Gd^3+^-MIH

Relaxometry and phantom studies were conducted to evaluate the enhancement of magnetic resonance imaging (MRI) capabilities of the MCM-41-NH_2_-DTPA-Gd^3+^-MIH nanomaterial. The purpose of these studies was to assess and evaluate the potential of the nanomaterial as a contrast agent by determining the effect of the gadolinium present in the nanomaterial on the MRI signal intensity.

**Figure 7:**
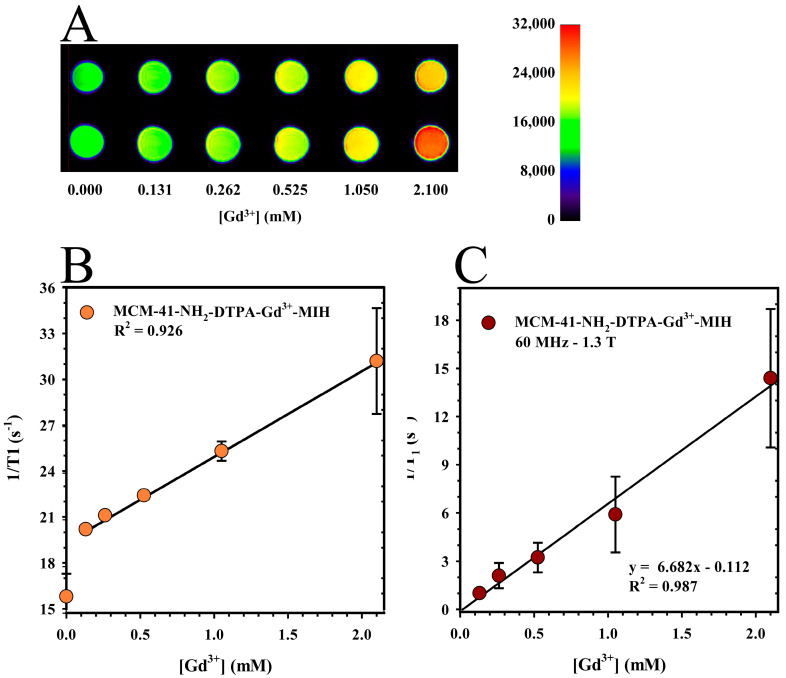
(**A**) T_1_-weighted magnetic resonance imaging (MRI) image of the MCM-41-NH_2_-DTPA-Gd^3+^-MIH nanomaterial at various Gd^3+^ concentrations in DIUF water and (**B**) linear fitting of the normalized voxel values of 2D ROIs as a function of Gd^3+^ concentration in DIUF water (●). Each data point represents the mean ± standard deviations from two duplicate phantoms. (**C**) Relaxation rate of MCM-41-NH_2_-DTPA-Gd^3+^-MIH (1/T_1_) at 1.3 T (●). Each data point represents the mean ± standard deviation of three replicates.

The MRI phantom studies utilized 0.5 mL microcentrifuge tubes as phantoms to evaluate the MRI capabilities of the MCM-41-NH_2_-DTPA-Gd^3+^-MIH nanomaterial. Figure 7A illustrates the T1-weighted MRI images, with increasing signal intensity represented by a progressive change in the color spectrum. In addition, a linear fit of normalized voxel values from 2D regions of interest (ROIs) as a function of Gd^3+^ concentration was performed, as shown in Figure 7B. These results demonstrated a strong concentration-dependent signal effect, with a coefficient of correlation (R^2^) of 0.996. Statistical analysis of the phantom data involved a paired *t*-test to compare voxel values at each level of gadolinium concentration, revealing a *p*-value greater than 0.05. This result signifies no statistically significant difference between the sets of voxel values, underscoring the consistency and reliability of the measurements across different gadolinium concentrations.

Relaxometry studies were performed to quantitatively measure the longitudinal relaxation rates (1/T_1_) of the MCM-41-NH_2_-DTPA-Gd^3+^-MIH nanomaterial, providing data into its effectiveness as an MRI contrast agent by assessing how it influences the relaxation times of water protons in its vicinity. As shown in Figure 7C, the results displayed a strong linear correlation between the longitudinal relaxation rate (1/T_1_) and the Gd^3+^ concentration. A linear regression analysis achieved a coefficient of determination (R^2^) of 0.992. This strong correlation highlighted the significantly enhanced longitudinal relaxivity (r_1_) of the nanomaterial at 6.7 s^−1^·mM^−1^. In comparison, the commercially available MRI contrast agent, Magnevist (Gd-DTPA), had an r_1_ of approximately 4.3 s^−1^·mM^−1^ at 1.5 Tesla [68]. Such a notable increase in relaxivity suggests that MCM-41-NH_2_-DTPA-Gd^3+^-MIH is a superior contrast agent, potentially offering improved clarity and detail in MRI imaging.

### 2.8. In Vivo Imaging

In vivo, imaging studies were performed to assess the distribution and efficacy of the MCM-41-NH_2_-DTPA-Gd^3+^-MIH nanomaterial as a contrast agent in tumor-bearing BALB/c mice. In these experiments, T1-weighted MRI scans were obtained before and 15 min after an intratumoral injection of the nanomaterial. We performed intratumoral injection of the nanomaterial to provide physical delivery of the theranostic agent directly to the tumor site, thereby maximizing the local concentration and minimizing systemic exposure. This approach allowed for a more accurate assessment of the contrast effect in a in a specific location. The tumor regions, highlighted by red circles in the images, showed significant contrast enhancement post-injection compared to pre-injection. This signal increase demonstrated the efficacy of the nanomaterial as a contrast agent for tumor imaging.

## 3. Discussion

The physicochemical characterizations performed on the MCM-41 nanomaterial showed the successful formation and functionalization of the nanomaterial. The SEM image (Figure 1A) confirmed the uniformity in the synthesized nanoparticles, a crucial attribute for various applications, including drug delivery, where a size around 60 nm with uniformity is essential for consistent release profiles [69]. These results align with the notion that the synthesis conditions play a vital role in dictating the final characteristics of the nanoparticles. From the TEM images (Figure 1B), the formation of a nanomaterial with a highly ordered pore structure was observed, characteristic of the MCM-41-type silica [70].

Uniformity in particle size around 60 nm is critical, as discussed by Park et al. [71], who noted that size uniformity directly impacts the biodistribution and clearance rates from the body. This improvement could lead to more predictable pharmacokinetics and pharmacodynamics in clinical applications. For example, this ordering is crucial for applications where regularity and pore size control are essential, such as in the adsorption of drugs like MIH 2.4Bl. It is also possible to observe (in Figure 1B) the presence of uniform, well-defined cylindrical pores, suggested by the consistency in the contrast of dark regions (the pores) relative to light regions (the silica walls). This characteristic feature facilitates drug diffusion and allows for controlled and targeted release, a highly advantageous attribute in chemotherapy [46,51,52].

The FTIR spectra (Figure 2A) displayed prominent absorption bands corresponding to the CTAB surfactant before calcination. The disappearance of these bands post-calcination signified the effective removal of the surfactant, a crucial step for enabling subsequent drug adsorption. As demonstrated by the spectral changes, this change supported the successfully creation of a surfactant-free mesoporous silica structure. The porosimetry analysis (Figure 2B) confirmed a significantly larger surface area and pore volume than those reported by Shariatinia and Pourzadi [47]. Such characteristics are beneficial not only for high drug load capacity, but also for increased interaction with biomolecules, which is critical for applications in drug delivery and catalysis. The specific pore size of 3.7 nm, ideal for various biomedical applications, aligns with findings by Alexis et al. [69], who emphasized the importance of pore size in controlling drug release rates.

The emergence of specific bands in the FTIR spectrum post-functionalization with APTES (Figure 3A) indicated the successful incorporation of amine groups on the silica surface. The formation of Ruhemann’s purple when the ninhydrin reagent reacted with the primary amines (Figure 3B) confirmed this modification, which was essential for subsequent conjugation steps with DTPA. Thus, this modification enhances the utility of the nanomaterial in targeted applications such as imaging and provides a pathway for further functionalization [72].

FTIR spectra of the MCM-41-NH_2_-DTPA nanomaterial (Figure 4A) indicated the successful conjugation of DTPA to the silica surface, a modification pivotal for the complexation with gadolinium ions, enhancing its application as a contrast agent in MRI [64]. The coordination of Gd^3+^ ions to DTPA, indicated by shifts in FTIR frequencies, demonstrates the effective complexation essential for diagnostic imaging applications. In addition, the successive decrease in Gd^3+^ concentrations over successive washes (Figure 4B) were critical for confirming the chelation efficacy.

The rapid initial adsorption followed by a gradual increase to equilibrium (Figure 5A) indicated a high affinity of the MCM-41-NH₂-DTPA-Gd^3^⁺ nanomaterial for the drug MIH 2.4Bl. The stabilization of adsorption capacity at 6 h highlighted the optimal time for achieving maximum drug loading. The optimized adsorption kinetics of the MCM-41-NH₂-DTPA-Gd^3^⁺ nanomaterial for the drug MIH 2.4Bl demonstrated a significant improvement over those documented by Jia et al. [66,73], where lower drug-loading capacities were observed for paclitaxel. Subsequent ICP-OES characterization confirmed the presence of Gd^3+^ in the nanomaterial and its suitability for biomedical applications, particularly in diagnostic imaging.

In the drug release assays, there was an initially rapid release phase followed by a slower phase, which could be explained in terms of the diffusion and interactions of the drug with the MCM-41-NH_2_-DTPA-Gd^3+^ nanomaterial. The drug diffusion may have been influenced by the porosity of the nanomaterial and the strength of the interactions between the drug and the nanomaterial. Nonetheless, controlled drug release after the initial peak may be beneficial in therapeutic applications, providing more sustained delivery that can help maintain drug concentrations within a desired therapeutic range without the need for frequent administration.

We noted that the EC_50_ for the MDA-MB-231 cells was significantly lower compared to HMECs, demonstrating selective cytotoxicity towards cancer cells. The observed EC_50_ differences between MDA-MB-231 breast cancer cells and HMECs can be attributed to several factors. First, MDA-MB-231 cells are cancerous and exhibit different metabolic activities and drug sensitivities than normal HMECs [74]. In addition, we have previously shown that the cytotoxic activity of the mesoionic compound MIH 2.4Bl is more pronounced in breast cancer cells, likely due to their higher proliferative rate and altered biochemical pathways [24,27]. Finally, MSNs are taken up by cells through endocytosis, a process that involves the engulfment of nanoparticles by the cell membrane to form endosomes. Cancer cells have been reported to possess an altered and enhanced capacity for endocytosis compared to normal cells [75]. This increased endocytic activity facilitates greater internalization of the MSNs, resulting in higher intracellular concentrations of the drug and enhanced cytotoxic effects. This is consistent with findings from other studies that have demonstrated enhanced nanoparticle uptake in cancer cells due to their elevated endocytic activity [76,77].

The cytotoxicity of MCM-41-NH_2_-DTPA-Gd^3+^-MIH nanoparticles against breast cancer cells is consistent with findings from other studies involving MSNs loaded with therapeutic agents, such as doxorubicin [78] and paclitaxel [79]. These studies have also shown that MSNs can effectively deliver drugs to cancer cells while minimizing toxicity to normal cells due to their high surface area, pore volume, and ability to be functionalized for targeted delivery. Our results reinforce the role of MSNs in enhancing the efficacy and safety profile of chemotherapeutic agents.

**Figure 8:**
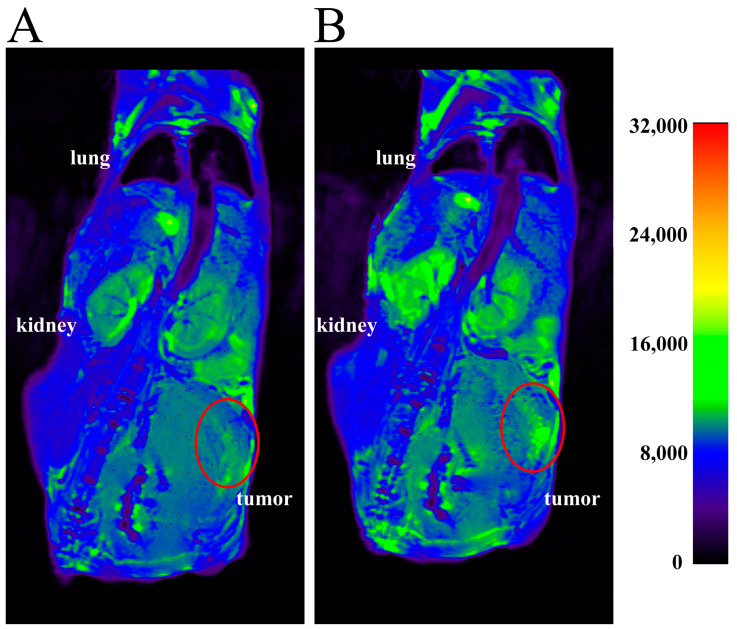
Representative MRI scans of BALB/c mice with 4T1 tumors (**A**) before and (**B**) at 15 min after intratumoral injection with MCM-41-NH_2_-DTPA-Gd^3+^-MIH nanomaterial. The scans show the T_1_-weighted MRI signal intensity in various tissues. The tumor regions are highlighted by the red circles.

The MRI phantom studies underscored the capacity of the MCM-41-NH_2_-DTPA-Gd^3+^ nanomaterial to enhance MRI signal intensity in a Gd^3^⁺ concentration-dependent manner (Figure 7A). The consistent linearity in voxel values as Gd^3^⁺ concentration increased (Figure 7B) reaffirms the efficacy of the nanomaterial as a promising MRI contrast agent. The *t*-test results, with a *p*-value greater than 0.05, confirmed the high consistency and precision of the measurements, crucial for diagnostic applications. The linearity observed in voxel values with increasing Gd^3^⁺ concentrations underscore the potential of the nanomaterial as a contrast agent. The relaxometry results (Figure 7C) also demonstrated a strong linear correlation between the longitudinal relaxation rate (1/T_1_) and the concentration of Gd^3^⁺ ions. In addition, the r_1_ value of the MCM-41-NH₂-DTPA-Gd^3^⁺-MIH nanomaterial indicated a significantly superior efficacy of the compound compared to currently available commercial magnetic resonance imaging (MRI) contrast agents [68,80]. The performance of the nanomaterial suggests not only an enhanced signal intensity, but also a potential reduction in the amount of contrast agent required for effective imaging, addressing some of the key limitations in current MRI contrast agent formulations, such as nephrogenic and systemic toxicity. The results of the in vivo MRI studies in tumored mice (Figure 8) reinforce the potential of MCM-41-NH_2_-DTPA-Gd^3+^-MIH as an MRI contrast agent for breast cancer imaging and support further investigation and development. 

The Enhanced Permeability and Retention (EPR) effect [81] allowed for preferential accumulation within the tumor site upon intratumoral administration of MSNs. When MCM-41-NH_2_-DTPA-Gd^3+^-MIH was administered intratumorally, the EPR effect ensured these nanoparticles were retained within the tumor microenvironment for extended periods. This prolonged retention increased the likelihood of cellular uptake by tumor cells. The mechanism of cellular uptake of MSNs also played a crucial role in their targeting efficacy. MSNs are primarily taken up by cells through endocytosis, a process that involves the engulfment of nanoparticles by the cell membrane to form endosomes [77]. This uptake mechanism is influenced by several factors, including particle size and surface charge. For example, MSNs with a size range of 50 to 200 nm are optimal for cellular uptake, as they are efficiently internalized by cells while avoiding rapid clearance by the immune system. In addition, positively charged MSNs tend to have higher cellular uptake rates due to electrostatic interactions with the negatively charged cell membrane.

Overall, this work indicates that the synthesized MCM-41-NH₂-DTPA-Gd^3^⁺-MIH nanomaterial has the potential for further development in treating breast cancer, particularly as a dual drug delivery and diagnostic imaging agent. Despite the promising results, our study has several limitations that will be addressed in future studies. While the MCM-41-NH_2_-DTPA-Gd^3+^-MIH demonstrated potential as an effective MRI contrast and therapeutic agent, the current construct lacks specific targeting ligands. Future experiments will be designed to enhance the specificity of the MCM-41-NH_2_-DTPA-Gd^3+^-MIH towards tumor cells by the incorporation of targeting ligands or antibodies that recognize and bind to overexpressed receptors such as the folate receptor on the surface of breast cancer cells [82]. While preliminary in vivo studies showed enhanced imaging capabilities, comprehensive in vivo efficacy and safety studies are needed. Future in vivo studies will focus on further validating the imaging capabilities of the nanomaterial, involving longitudinal imaging to monitor the biodistribution and tumor uptake via different routes of administration (such as intravenous). In addition, the therapeutic efficacy will be assessed by evaluating tumor regression and overall survival rates in mice treated with the nanomaterial compared to control groups. In vivo studies will be performed to assess any off-target effects in non-target organs and determine the immune response to the nanomaterial. While the study provided insights into the drug release profile, the kinetics were performed under in vitro conditions. Future studies will focus on optimizing the drug release kinetics in vivo to provide sustained release of the MIH 2.4Bl drug at the tumor site.

## 4. Material and Methods

### 4.1. Materials and Reagents

Cetyltrimethylammonium bromide (CTAB), tetraethyl orthosilicate (TEOS), 3-Cetyltrimethylammonium bromide (CTAB), tetraethyl orthosilicate (TEOS), 3-aminopropyltriethoxysilane (APTES), ninhydrin, diethylenetriaminepentaacetic dianhydride (DTPA), dimethylformamide (DMF), gadolinium hexahydrate (GdCl_3_·6H_2_O), phosphate-buffered saline (PBS), Dulbecco’s Minimum Essential Medium (DMEM), RPMI 1640 Medium, as well as dialysis bags and Amicon^®^ Ultra-4 Centrifugal Filters were acquired from MilliporeSigma (Burlington, MA, USA). The drug 2-(4-chlorophenyl)-3-methyl-4-(4-methyl phenyl)-1,3-thiazolium-5-thiolate (MIH 2.4Bl) was obtained through a partnership with the Bioenergy and Organic Synthesis Research Laboratory (LPBS), located in the Chemistry Department (DQ) of the Center for Exact and Natural Sciences (CCEN) at the Federal University of Paraíba (UFPB), João Pessoa campus (João Pessoa, PB, Brazil). Reagents including ammonium hydroxide (NH_4_OH), anhydrous ethanol, 97% ethanol, sodium hydroxide (NaOH), and hydrogen peroxide (H_2_O_2_) were obtained from Dinâmica Química Contemporânea (Indaiatuba, SP, Brazil). Solutions of antibiotics, amino acids, HEPES, trypsin/EDTA, and trypan blue dye were obtained from Thermo Fisher Scientific (Waltham, MA, USA).

### 4.2. Synthesis of Mesoporous Silica (MCM-41)

The mesoporous silica nanoparticles were prepared using the method developed in the literature [52]. A total of 442.0 mL of ultrapure water and 2.0 mL of NH_4_OH (29%) were added to a 1.0 L Erlenmeyer flask under magnetic stirring at 800 rpm and heating at 80 °C. Subsequently, 0.279 g of CTAB was introduced into the flask, which was maintained under constant magnetic stirring and heating conditions for 3 h. Afterward, the system was cooled to room temperature, and 1.39 mL of TEOS was added slowly. The mixture was maintained under stirring for 2 h. Then, the formed nanomaterial was washed three times with ethanol and dried in a vacuum oven at 50 °C for 24 h. The formed MCM-41 silica nanoparticles were then calcined for a period of 4 h at 550 °C with a heating ramp of 5 °C/min and ground for subsequent functionalization with APTES silane.

### 4.3. Functionalization of MCM-41 with APTES (MCM-41-NH_2_)

The mesoporous silica nanoparticles were functionalized using a method based on the literature [83]. To a round-bottom flask under reflux at 80 °C, 50.0 mL of anhydrous ethanol and 0.150 g of MCM-41 were added. The reaction conditions were maintained under constant magnetic stirring at 400 rpm for 5 min. Subsequently, APTES (2.5 mL) was slowly added dropwise, and the reaction was kept under reflux for 24 h. Afterward, the reaction mixture was cooled to room temperature, and the resultant nanomaterial (MCM-41-NH_2_) was washed with ethanol three times and dried in a vacuum oven for 12 h at 60 °C. For qualitative confirmation of the nanoparticle functionalization with APTES [84], an evaluation was conducted using the ninhydrin reagent. The ethanolic suspensions of MCM-41 and MCM-41-NH_2_ nanomaterials (10 mg/5 mL ethanol) were prepared in two separate containers. Subsequently, five drops of a 2% ethanolic ninhydrin solution were added to each suspension. Finally, the mixtures were heated in an ultrasonic bath at 37 °C for 15 min, and a colorimetric change of the nanomaterials suspensions was observed to confirm the functionalization of MCM-41 with APTES.

### 4.4. Conjugation of MCM-41-NH_2_ with Diethylenetriaminepentaacetic Dianhydride (DTPA)—(MCM-41-NH_2_-DTPA)

The nanoparticles were surface-conjugated with the chelating agent DTPA [85]. Quantities of 55.0 mg of MCM-41-NH_2_ and 9.5 mL of anhydrous DMF were added to a round-bottom flask. The mixture was sonicated for 15 min and then heated to 70 °C under reflux conditions. Subsequently, 28.0 mg of DTPA was added to the flask. The reaction was maintained under magnetic stirring at 800 rpm and heated at 70 °C for 20 h. Finally, the formed MCM-41-NH_2_-DTPA nanomaterial was washed several times with DMF and distilled water, purified using an Amicon^®^ Ultra-4 Centrifugal Filter, and dried in a vacuum oven at 40 °C for 24 h.

### 4.5. Complexation of MCM-41-NH_2_-DTPA with Gadolinium (Gd^3+^)—(MCM-41-NH_2_-DTPA-Gd^3+^)

A volume of 5.0 mL of gadolinium chloride hexahydrate (GdCl_3_·6H_2_O) solution at a concentration of 0.07 mM was added to a round-bottom flask containing 40.0 mg of the MCM-41-NH_2_-DTPA nanomaterial. The mixture was maintained for 48 h under magnetic stirring (1000 rpm) at 37 °C. The pH was monitored throughout the process and adjusted to between 6.0 and 7.0 using a sodium hydroxide solution (1.0 M). After 48 h, the resulting nanomaterial was washed several times with distilled water, purified with the help of an Amicon^®^ Ultra-4 Centrifugal Filter, and dried in a vacuum oven (40 °C) for 24 h. To evaluate the stability of gadolinium complexed to the DTPA ligand, aliquots from each washing were analyzed, and the ion concentration was determined using Inductively Coupled Plasma Optical Emission Spectrometry (ICP-OES) system (PerkinElmer, Inc.; Waltham, MA, USA) as previously described by Ratanajanchai et al. [86].

### 4.6. Adsorption of MIH 2.4Bl Drug to MCM-41-NH_2_-DTPA-Gd^3+^Nanomaterial (MCM-41-NH_2_-DTPA-Gd^3+^-MIH)

A quantity of 20.0 mg of MCM-41-NH_2_-DTPA-Gd^3+^ (adsorbent) was added to a glass vial containing 2 mL of MIH 2.4Bl solution dissolved in methanol (3.0 mM adsorbate). The adsorbent and adsorbate were maintained under magnetic stirring (1000 rpm) at room temperature. Subsequently, the mixture was centrifuged, and the supernatant was discarded. The resultant nanomaterial was then dried at room temperature. The kinetic study of adsorption was conducted using 2 mL of the adsorbate at a concentration equivalent to 3.0 mM and 20 mg of the adsorbent nanomaterial. The adsorbent/adsorbate system was kept under constant agitation (1000 rpm) at room temperature (26 °C) using a model 5355 Eppendorf Thermomixer (Eppendorf Scientific; Hamburg, Germany). The suspensions were centrifuged periodically from 1 to 48 h, and their concentrations were determined [87] by measuring changes in absorbance at 415 nm using a UV-Vis spectrophotometer (Shimadzu UV-1650; Kyoto, Japan).

Using Equation (1), the adsorption capacity at times was calculated based on the difference between the initial and final concentrations of the drug in the solution.

(1) $$q_{t\;}=\frac{\left(C_{0}-C_{t}\right)\cdot \;V}{m}$$

where:

- *q_t_* is the adsorption capacity, or the amount of drug adsorbed per unit mass of the adsorbent at time *t*, given in mg/g.
- *C*_0_ (mg/L) is the initial concentration of the adsorbate.
- *C_t_* is the adsorbate concentration at time *t*, given in mg/L.
- *m* is the mass of the adsorbent, given in grams (g).
- *V* is the volume of the solution, given in liters (L).

### 4.7. Drug Release of the Mesoionic Drug MIH 2.4Bl Adsorbed to MCM-41-NH_2_-DTPA-Gd^3+.^

The release study of the MIH 2.4Bl drug adsorbed to MCM-41-NH_2_-DTPA-Gd^3+^ was conducted using the dialysis membrane technique based on the methodology described by Fuller et al. [88], with a dialysis medium composed of a 75:25 *v*/*v* mixture of phosphate-buffered saline (PBS; pH 7.4) and methanol. Saturation conditions were maintained throughout the experiment. For the study, 10 mg of MCM-41-NH_2_-DTPA-Gd^3+^-MIH nanomaterial was suspended in 1.0 mL of medium and added to the dialysis membrane. The dialysis membranes were then immersed in 6 mL of medium, and the systems were maintained under constant magnetic stirring at 37 °C. Aliquots of 1.0 mL were collected at pre-determined times (0.5, 1, 3, 6, 12, 24, and 48 h) and immediately replaced with fresh medium. The concentrations of each aliquot were determined by measuring the absorbance at 415 nm using a UV-Vis spectrophotometer (Shimadzu UV-1650; Kyoto, Japan).

### 4.8. Material Characterizations

#### 4.8.1. Fourier Transform Infrared Spectroscopy with Attenuated Total Reflectance (ATR-FTIR)

For mapping the structure of the compounds and verifying the presence of functional groups in their constituents, the MCM-41, MCM-41-NH_2_, MCM-41-NH_2_-DTPA, and MCM-41-NH_2_-DTPA-Gd^3+^ nanomaterials were analyzed using ATR-FTIR as previously described by Laprise-Pelletier et al. [44]. The spectra were obtained using a Nicolet iS5 FTIR spectrophotometer (Thermo Fisher Scientific) through the reflectance method in the range of 4000–550 cm^−1^, with a precision of 0.5 cm^−1^ and 32 accumulations. The powders of all samples were deposited directly onto the crystal for subsequent reading.

#### 4.8.2. Scanning Electron Microscope (SEM)

The morphologies of the nanomaterials MCM-41 were evaluated using a TESCAN MIRA 3 SEM instrument (TESCAN GROUP, a.s.; Brno, Czech Republic) with a voltage of 10 kV. The sample was fixed on a sample holder with the help of a carbon tape, and then it was metalized with 10 nm of gold for 10 min using an SC-701 metallizer (Sanyu Electronics Co., Ltd.; Tokyo, Japan). Subsequently, the sample holder was inserted into the SEM instrument, and the sample was then characterized. Subsequently, the acquired images were analyzed [89]. The ImageJ software (version 1.53k, created by Wayne Rasband, National Institutes of Health, Bethesda, MD, USA) was used to facilitate the calculation of the average size and diameter of the nanoparticles based on the images obtained by SEM.

### 4.9. Inductively Coupled Plasma Optical Emission Spectrometry (ICP-OES)

ICP-OES was used to quantitatively evaluate the presence of gadolinium in the MCM-41-NH_2_-DTPA-Gd^3+^ and MCM-41-NH_2_-DTPA-Gd^3+^-MIH nanomaterials, as previously described [44]. For the analysis, the samples were initially digested in a 5% nitric acid and 30% hydrogen peroxide solution at a temperature of 115 °C for 30 min to ensure complete decomposition of the samples into their constituent elements. After digestion, the resulting solutions were carefully introduced into an ICP-OES Optima 7000 DV instrument (PerkinElmer, Inc.; Waltham, MA, USA). The instrument was then used to acquire spectrometric data on the gadolinium content in each sample.

### 4.10. Porosimetry

The surface area of the MCM-41 nanomaterial was determined through nitrogen adsorption/desorption (77 °K) using the Brunauer, Emmett, and Teller (BET) method [90]. This analysis was conducted using a Micromeritics ASAP 2020 Microporosimeter (Micromeritics Instrument Corporation; Norcross, GA, USA). Before the BET analysis, a 0.1 g sample of MCM-41 was prepared by drying it in an oven for 12 h at 100 °C. This step was crucial in order to eliminate any moisture that could affect the precision of the surface area measurement. After the drying process, the sample was then subjected to porosity analysis to assess its surface area.

### 4.11. Zeta Potential

The zeta potential values of the nanoparticles MCM-41, MCM-41-NH_2_, and MCM-41-NH_2_-DTPA were measured using a Zetasizer Nano zeta potential analyzer (Malvern Instruments Ltd., Malvern, UK) at 25 °C, with ultra-pure water as the solvent.

### 4.12. Transmission Electron Microscopy (TEM)

The MCM-41 nanomaterial was analyzed using TEM to identify the average size and diameter of the nanoparticle [44]. A JEOL- 2100 TEM (JEOL, Ltd.; Tokyo, Japan) operating at 200 kV was utilized for this analysis. The material was dispersed in EtOH and then deposited on a carbon-coated copper grid. The grid was then dried under a vacuum before analysis.

### 4.13. MRI Phantom Studies

Through MRI phantom analysis, contrast images were obtained, which were essential for evaluating the efficacy of the contrast agent MCM-41-NH_2_-DTPA-Gd^3+^-MIH across a range of concentrations of Gd^3+^. Colloidal suspensions of this agent were prepared in five distinct concentrations using ultrapure water and then inserted into an MRS*DRYMAG 7T magnetic resonance imaging instrument (MR Solutions; Guildford, UK). This instrument operated at a Larmor frequency of approximately 297.2 MHz, corresponding to a magnetic field of 7 Tesla. A 31 mm diameter volumetric coil was used to optimize field homogeneity and signal sensitivity. For acquiring T_1_-weighted images, the following technical parameters were set.

- Field of view (FOV): 75/37.5 cm, providing adequate sample coverage.
- Slice thickness: 1 mm to ensure optimal spatial resolution.
- Repetition time (TR): 1000 ms, adjusted to optimize contrast between different tissues.
- Echo time (TE): 11 ms, chosen to minimize signal loss due to magnetic field inhomogeneities.
- Number of slices: 12, allowing detailed visualization of the sample in multiple sections.
- Flip angle: 90° to maximize the received signal.
- Number of averages: 7, aiming to improve the signal-to-noise ratio.
- Slice gap: 0.2 mm to avoid overlap or gaps between slices.
- Total acquisition time: 14 min, balancing the need for high-quality imaging with time efficiency.

After the acquisition, the images were processed and visualized using 3D Slicer software (3D Slicer version 5.6.2, created by the Surgical Planning Laboratory at the Brigham and Women’s Hospital and the MIT Artificial Intelligence Laboratory, Boston, MA, USA) [91]. Image analysis and pixel quantification of regions of interest (ROIs) were performed using AMIDE software (AMIDE version 1.0.6, created by Andy Loening and Sanjiv Sam Gambhir, Stanford University, Stanford, CA, USA) [92] to comparatively evaluate the nanomaterials in terms of the distribution and intensity of the signal.

### 4.14. Relaxometry

A relaxometry characterization of the MCM-41-NH_2_-DTPA-Gd^3+^-MIH nanomaterial was conducted to evaluate the potential as contrast agents in magnetic resonance imaging (MRI), as carried out in the study cited in [44]. Colloidal suspensions in five distinct concentrations of Gd^3+^ were prepared to assess the relaxivity dependence of the longitudinal (T_1_) relaxation time on the concentration of the nanomaterial. The measurements were directed at the hydrogen (^1^H) nuclei present in the water molecules of the suspensions. The principle of the measurements lies in analyzing the T_1_ relaxation time to evaluate the efficacy of the nanomaterials as contrast agents in MRI applications. The experiment was conducted using a Minispec mq60 instrument (Bruker; Billerica, MA, USA) operating in a magnetic field of 1.41 Tesla (60 MHz). Measurements were performed at a controlled temperature of 37 °C, simulating the human physiological environment.

### 4.15. Biological Characterizations

#### 4.15.1. Cell Culture

In vitro biological assays were conducted using human breast cancer cells and normal mammary epithelial cells (HMECs). The MDA-MB-231 human breast cancer cell line obtained from ATCC (Manassas, VA, USA) was cultured in RPMI-1640 medium (MilliporeSigma; Burlington, MA, USA) supplemented with 2.5% HEPES, 1% antibiotic solution, 1% amino acids, and 10% fetal bovine serum (FBS; Gemini Bio-Products; Sacramento, CA, USA). Human Primary Mammary Epithelial Cells (H-6035) were obtained from Cell Biologics, Inc. (Chicago, IL, USA) and cultured in collagen-coated tissue culture flasks using Gelatin-Based Coating Solution (H6950); Cell Biologics) with complete Human Epithelial Cell Medium (H6621; Cell Biologics), All cells were maintained at 37 °C in a 5% CO_2_ humidified incubator and passaged before reaching 90% cell confluency. Before each experiment, cells were counted by combining 10 µL of cell suspension with 90 µL of trypan blue, and the cell number was determined manually using a hemocytometer.

#### 4.15.2. Cell Viability Assay

An MTT cell viability assay [93] was performed to evaluate the cytotoxic effects of the synthesized nanoparticles (MCM-41-NH_2_-DTPA-Gd^3+^ and MCM-41-NH_2_-DTPA-Gd^3+^-MIH) loaded with the mesoionic drug MIH 2.4Bl on the MDA-MB-231 breast cancer cell line and normal human mammary epithelial cells (HMECs). The cells were seeded in a 96-well plate at 2000 cells/well and incubated with the nanoparticles at increasing concentrations from 3.9 µg/mL media to 250 µg/mL media for 96 h. Cell viability was determined by adding 10 µL of MTT (5 mg/mL) dye and incubation for 3 h, followed by adding 100 µL stop solution to solubilize the formazan crystals. The absorbance of each well was measured at 570 nm using a Multiskan SkyHigh microplate spectrophotometer (Thermo Scientific). The experiments were conducted in triplicate, and the results were calculated relative to the untreated cells designated as 100% viable. Percent viability was normalized to the maximum and minimum absorbance values.

#### 4.15.3. Injection of 4T1 Cells in BALB/c Mice

The murine breast cancer 4T1 cell line was obtained from ATCC (Manassas, VA, USA) and cultured in RPMI-1640 medium enriched with 2.5% HEPES, 1% antibiotic, 1% amino acids, and 10% fetal bovine serum (FBS). The cells were inoculated into ten T-75 flasks containing 25 mL of culture medium, with a density of 5 × 10^4^ cells per flask. The flasks were incubated at 37 °C in a 5% CO_2_ atmosphere for 4 days, with medium renewal every 48 h. After this period, the cells were washed with 1% PBS (pH 7.4), detached using a trypsin/EDTA solution, and collected by centrifugation at 1000 rpm for 4 min. Cell viability was assessed using 50% trypan blue and quantified with a hemocytometer, resulting in a cell suspension concentration of 6 × 10^7^ cells/mL in PBS.

Female BALB/c mice, aged 7–8 weeks and weighing 25–30 g, were obtained from Taconic Biosciences, Inc. (Germantown, NY, USA). The research protocol was approved by the Institutional Animal Care and Use Committee (IACUC) of the University of North Texas Health Science Center in Fort Worth (protocol number 2023-0026) and followed the Guide for the Care and Use of Laboratory Animals [94]. Each mouse was subcutaneously inoculated with 100 μL of the cell suspension (6 × 10^6^ cells/mL in saline solution). The tumor growth was monitored daily after inoculation; in vivo, experiments began 14 days after tumor implantation when the tumors had reached an average diameter of approximately 10 mm.

#### 4.15.4. MRI Analysis in BALB/c Mice with Tumors

Magnetic resonance imaging (MRI) analysis in BALB/c mice aimed to evaluate the distribution and efficacy of the signal of the contrast agent MCM-41-NH_2_-DTPA-Gd^3+^-MIH in vivo in both normal mice and those with tumors. Initially, baseline MRI images were obtained without administering any contrast agents to serve as a comparative control. For administration, a colloidal suspension of MCM-41-NH_2_-DTPA-Gd^3+^-MIH was prepared at a concentration of 5 mg/mL in phosphate-buffered saline (PBS) (pH 7.4). A volume of 0.1 mL of these suspensions was injected directly into the tumors of the mice using 30 G needles attached to 1 mL insulin syringes. Anesthesia was induced in the animals with 5.0% isoflurane and maintained at 2.5% during image acquisition. Respiration was continuously monitored to maintain a respiratory rate of 50–60 breaths per minute.

Contrast-enhanced MRI images were acquired using an MRS*DRYMAG 7T (MR Solutions) magnetic resonance imaging instrument operating at a frequency of 297.2 MHz (7 Tesla) with a 31 mm volumetric coil. The imaging protocol consisted of the following steps:

- T_1_-weighted images (Tw1) were acquired before injection and 15 min after the intratumoral injection of the nanoparticles, with a field of view (FOV) of 100/50 cm, a slice thickness of 1 mm, a repetition time (TR) of 1000 ms, an echo time (TE) of 11 ms, 12 slices with a flip angle of 90°, 7 averages, and a slice gap of 0.2 mm.
- After the acquisition, the images were processed and visualized using 3D Slicer software and quantified using AMIDE software as described previously.

### 4.16. Statistical Analysis

All analyses of the drug release of MIH 2.4Bl, MRI phantom, MRI in BALB/c mice with and without tumors, as well as cell viability assays, were conducted at least in triplicate. The data are presented as mean ± standard deviation. The analyses were performed using OriginPro software (OriginPro 2018, version b9.5.1.195, OriginLab Corporation, Northampton, MA, USA) and Python programming language (Python 3.7 Software Foundation, Beaverton, OR, USA) specifically employing the SciPy statistical library [95]. Statistical comparisons between different groups of independent samples were made using the *t*-test. This test was performed through the “ttest_ind” function from the “scipy.stats” submodule, specifically designed to evaluate two independent variables. The function calculates t-statistics and *p*-values, considering the variances and sample sizes of the groups being compared. A *p*-value was calculated for each comparison to assess the statistical significance of the differences between the means of each pair of groups. The threshold for significance was set at 0.05; *p*-values above this level were interpreted as indicating no significant difference between the means of the compared groups.

## Figures and Tables

**Table 1:** Zeta potential of MCM-41, MCM-41-NH_2_, and MCM-41-NH_2_-DTPA.

Samples	Zeta Potential (mV)
MCM-41	−22.5 ± 0.499
MCM-41-NH_2_	+12.9 ± 0.386
MCM-41-NH_2_-DTPA	−16.1 ± 0.170

## Data Availability

The datasets used and/or analyzed during the present study are available from the corresponding author upon reasonable request.
